# Bacterial microbiota and proinflammatory cytokines in the anal sacs of treated and untreated atopic dogs: Comparison with a healthy control group

**DOI:** 10.1371/journal.pone.0298361

**Published:** 2024-05-30

**Authors:** Camylle C. Bergeron, Marcio Carvalho Costa, Mariela Segura, Lucilene Bernardi de Souza, Marêva Bleuzé, Frédéric Sauvé

**Affiliations:** 1 Department of Clinical Sciences, Faculty of Veterinary Medicine, Université de Montréal, Saint-Hyacinthe, Quebec, Canada; 2 Department of Veterinary Biomedical Sciences, Faculty of Veterinary Medicine, Université de Montréal, Saint-Hyacinthe, Quebec, Canada; 3 Department of Pathology and Microbiology, Faculty of Veterinary Medicine, Université de Montréal, Saint-Hyacinthe, Quebec, Canada; 4 Centre Hospitalier Universitaire Vétérinaire, Faculty of Veterinary Medicine, Université de Montréal, Saint-Hyacinthe, Quebec, Canada; IHRF / Studio Rinaldi, ITALY

## Abstract

The pathogenesis of anal sacculitis has not been extensively investigated, although atopic dogs seem to be predisposed to the disease. The aim of this study was therefore to characterize and compare the bacterial microbiota and pro-inflammatory cytokines in the anal sacs of dogs from three groups (healthy dogs, untreated atopic dogs and atopic dogs receiving antipruritic treatment or allergen-specific immunotherapy) in order to determine whether changes could be at the origin of anal sacculitis in atopic dogs. Bacterial populations of anal sac secretions from fifteen healthy dogs, fourteen untreated and six treated atopic dogs were characterized by sequencing the V4 region of the 16S rRNA gene using Illumina technology. Proinflammatory cytokines were analyzed with the Luminex multiplex test. Community membership and structure were significantly different between the anal sacs of healthy and untreated atopic dogs (*P* = 0.002 and *P* = 0.003, respectively) and between those of untreated and treated atopic dogs (*P* = 0.012 and *P* = 0.017, respectively). However, the community structure was similar in healthy and treated atopic dogs (*P* = 0.332). Among the proinflammatory cytokines assessed, there was no significant difference between groups, except for interleukin 8 which was higher in the anal sacs of untreated atopic dogs compared to treated atopic dogs (*P* = 0.02), and tumor necrosis factor-alpha which was lower in the anal sacs of healthy dogs compared to treated atopic dogs (*P* = 0.04). These results reveal a dysbiosis in the anal sacs of atopic dogs, which may partially explain the predisposition of atopic dogs to develop bacterial anal sacculitis. Treatments received by atopic dogs (oclacitinib, desloratadine and allergen-specific immunotherapy) shift the microbiota of the anal sacs towards that of healthy dogs. Further studies are required to identify significant cytokines contributing to anal sacculitis in atopic dogs.

## Introduction

Dogs have skin invaginations on either side of the anus, called anal sacs, which are located between the smooth muscle of the internal sphincter and the striated muscle of the external sphincter of the anus [1–3]. During defecation, the contents of the anal sac are evacuated via a duct that opens at the anocutaneous junction [2, 4]. The role of the anal sacs is uncertain, but they appear to be involved in olfactory communication in dogs [1]. Anal sacculitis is inflammation of the anal sac, with or without infection [1, 5]. This condition could affect up to 12.5% of domestic dogs [3, 6]. In the presence of bacterial infection, local or systemic antimicrobial therapy is recommended [1, 3, 4, 7, 8]. Known predisposing factors to anal sacculitis include obesity, constipation, anal laxity, and chronic diarrhea [1, 3, 7]. Several diseases have also been reported to predispose to the development of anal sacculitis, such as dysendocrinia and atopic dermatitis [3, 4, 7, 8]. However, the exact etiopathogenesis of anal sacculitis remains unclear [1, 9].

Canine atopic dermatitis is a genetically programmed chronic inflammatory and pruritic skin disease mainly associated with IgE antibodies to environmental allergens [10, 11]. This disease is estimated to affect up to 30% of domestic dogs [11]. To better understand the pathogenesis of this disease, several studies have evaluated the cutaneous bacterial microbiota and the cytokines released in the skin as well as in the peripheral blood of atopic dogs [12–20]. Cutaneous bacterial microbiota dysbiosis was reported in atopic dogs [13, 14]. Indeed, atopic dogs have a less diversified and rich bacterial microbiota compared to healthy dogs [13, 14]. It is not clear whether dysbiosis is a consequence or a cause of the disease [11].

Marked differences in peripheral blood and skin cytokine profiles have been reported between atopic and healthy dogs [12]. However, there are several divergent results [12]. For example, Nuttall *et al*. found no significant difference in the expression of interleukin (IL)-12p40 mRNA between lesional skin of atopic dogs, non-lesional skin of atopic dogs and skin of healthy dogs [15]. However, Schlotter *et al*. detected a greater expression of IL-12p40 mRNA in the non-lesional skin of atopic dogs compared to the lesional skin of atopic dogs [16].

With the rise of antibiotic resistance and the high prevalence of canine atopic dermatitis, a better understanding of the etiopathogenesis of anal sacculitis would help to find alternative treatments to antimicrobials or preventative measures [14, 21]. To the best author’s knowledge, no studies have evaluated the bacterial microbiota in the anal sacs of atopic dogs nor the cytokines in the anal sacs of healthy and atopic dogs.

The aims of this study were: 1) to characterize the bacterial microbiota of the anal sacs and rectum of atopic dogs receiving or not antipruritic drugs or allergen-specific immunotherapy (ASIT); 2) to evaluate proinflammatory cytokines in the anal sacs of atopic and healthy dogs; 3) and, to compare the bacterial microbiota and proinflammatory cytokine profile and concentrations in the anal sacs of untreated and treated atopic dogs and healthy dogs. The hypotheses were that the bacterial microbiota and proinflammatory cytokines in the anal sacs of healthy dogs would differ from those of atopic dogs treated or not with antipruritic drugs or ASIT, and that a difference would be observed between the two atopic dog groups.

## Material and methods

This study followed the rules of the Canadian Council on Animal Care and was approved by the Faculty of Veterinary Medicine of the Université de Montréal’s Animal Care Committee (Comité d’éthique de l’utilisation des animaux, project number 18-Rech-1964). Dogs enrolled in this study were initially from the MIRA Foundation [22]. The MIRA Foundation had signed a written consent form for participation in this study, as well as current owners of the disqualified dogs and no longer belonging to MIRA Foundation.

### Animal selection

Twenty dogs with atopic dermatitis were enrolled in this study. Six of them (n = 6) were treated with an antipruritic drug (oclacitinib or desloratadine) and/or ASIT, and fourteen were untreated. The control dogs consisted of fifteen healthy dogs of related breed. Table 1 shows the signalment of the dogs enrolled in this study.

**Table 1:** Signalment and treatment of enrolled dogs.

Enrolled dogs	Breed	Sex	Age (year)	Treatment
**Healthy dogs**				
1	Labernese	MN	2.5	None
2	Labernese	MN	1.2	None
3	Labernese	MN	1.0	None
4	Labernese	MN	1.0	None
5	Labernese	FS	1.2	None
6	Labernese	FS	1.2	None
7	Labrador Retriever	FS	11.8	None
8	Labernese	MN	1.0	None
9	Labernese	MF	1.3	None
10	Labernese	FF	3.7	None
11	Labrador Retriever	MN	4.6	None
12	Labernese	FF	4.3	None
13	Labernese	FF	3.5	None
14	Labernese	MN	1.0	None
15	Labernese	FS	3.0	None
**Untreated atopic dogs**				
16	Labrador Retriever	MN	4.6	None
17	Labernese	FS	5.2	None
18	Labrador Retriever	MN	3.6	None
19	Labernese	FS	4.2	None
20	Labernese	FS	3.5	None
21	Labernese	FS	3.5	None
22	Labrador retriever	FS	1.2	None
23	Labrador retriever	FS	1.3	None
24	Labernese	FS	1.3	None
25	Labernese	FS	1.3	None
26	Labernese	FS	2.1	None
27	Labernese	FS	1.3	None
28	Labernese	FF	1.7	None
29	Labernese	FS	1.0	None
**Treated atopic dogs**				
30	Labrador Retriever	FS	4.4	Apoquel 0.41 mg/kg once daily and ASIT
31	Labernese	FS	5.3	Apoquel 0.47 mg/kg once daily and ASIT
32	Labrador Retriever	FS	7.5	Apoquel 0.53 mg/kg once daily and ASIT
33	Labernese	FS	2.1	Apoquel 0.51 mg/kg once daily
34	Labrador Retriever	MN	3.6	Apoquel 0.41 mg/kg once daily
35	Labernese	MN	4.1	Desloratadine 10 mg (0,28 mg/kg) once daily and ASIT

MN, Male neutered; FS, Female spayed; MF, Male fertile; FF, Female fertile; ASIT, Allergen-specific immunotherapy

The diagnosis of atopic dermatitis was based on the exclusion of other causes of pruritus (cutaneous infection, parasitic infestation, flea bite hypersensitivity, food allergy) in dogs showing compatible clinical signs. Prior to enrollment in this study, a broad-spectrum antiparasitic treatment of a minimum duration of three months was administered and dogs were fed an eviction diet for eight weeks. An intradermal test was performed in all dogs diagnosed with atopic dermatitis.

Inclusion criteria consisted of an absence of clinical signs consistent with cancer, skin infection, and systemic diseases on physical examination. All dogs should not have received any topical treatment (antimicrobial, bath) within thirty days prior to the study nor have received a systemic antibiotic within three months prior to the study. In the treated atopic group, dogs were allowed to receive oclacitinib, lokivetmab, glucocorticoids, cyclosporine, antihistamine, or ASIT.

### Sample selection

Five samples per dog were collected on the same day, including three sterile flocked swabs (FLOQSwabs®, Murrieta, California, USA) from the rectum, right anal sac, and left anal sac, and two sterile microtubes containing secretions from each anal sac. All samples were collected between November 2018 and October 2021. The perianal area was first cleaned with sterile gauze soaked in 4% chlorhexidine (DermaChlor^TM^ 4, Dechra, Pointe-Claire, Quebec, Canada) to dislodge organic material. After waiting two minutes, a sterile flocked swab was then gently inserted two cm into the rectum to collect a sample of material from the rectal mucosa. A pair of sterile gloves was then put on and sterile lubricant was applied to the index finger. The index finger was then gently inserted into the rectum and the contents of the left anal sac were expelled by pressing the anal sac with the index finger into the rectum and the thumb onto the skin covering the anal sac. A sample of the secretions from the left anal sac was collected at the opening of the left anal sac duct with a sterile flocked swab (the first few drops were not collected so that the sample would be representative of the anal sac contents). When possible, the remaining contents of the left anal sac were then collected in a sterile microtube. The perianal area was again cleaned with sterile gauze soaked in 4% chlorhexidine, then the same process, wearing new sterile gloves, was performed with the contralateral anal sac. The microtubes were then centrifuged for ten minutes, and the supernatant was transferred to a new sterile microtube. All samples were frozen at -80°C until DNA extraction or use of Luminex xMAP technology.

### DNA extraction and sequencing

The commercial kit DNeasy PowerSoil (Qiagen, Hilden, Germany) was used to extract DNA from all samples taken with a sterile flocked swab, as well as four unused sterile flocked swabs (negative controls). The first step for DNA extraction was to cut the tip of the swabs and put them in a tube containing beads (one swab for each tube). The tubes were then vortexed. To allow lysis of the cells, a solution from the kit was then added to the tube. Subsequent steps were followed as recommended by the manufacturer [23]. The V4 hypervariable region of the bacterial 16S ribosomal RNA gene was then amplified by polymerase chain reaction with the primers 515 (forward) and 806 (reverse). The sequencing was done with the Illumina MiSeq IEMFile version 4 platform at the Genome Quebec McGill Innovation Centre. The V2 reagents kit (2 x 250 cycles) was used for sequencing.

Bioinformatic analyses were performed using mothur software following the standard operating procedure as recommended by Kozich *et al*. [24]. Alpha diversity assessing the number of different bacterial genera present in a community (richness), and their relative abundances (evenness) was investigated using the Chao index (richness) and the Simpson, as well as the Shannon indices (diversity). Beta diversity measures were used to compare community similarities between samples by using the Jaccard index, a measure of the community membership that considers only the presence or absence of each genus, and the Yue and Clayton index, a measure of community structure that considers the relative abundance of each genus.

### Assessment of anal sac proinflammatory cytokines

Fluid samples from dogs’ anal sacs were thawed at room temperature. For each dog, 12.5 μL was taken from each anal sac (right and left) and mixed by vortex to obtain a total volume of 25 μL. These 25 μL samples per dog were used to measure the concentration of various proinflammatory cytokines using the Canine Cytokine/Chemokine/Growth Factor 11-Plex ProcartaPlex Panel (Invitrogen, Burlington, ON, Canada): interferon-gamma (IFN-γ), IL-10, IL-12/IL-23p40, IL-2, IL-6, IL-8, monocyte chemoattractant protein-1 (MCP-1), nerve growth factor beta (NGF-β), stem cell factor (SCF), tumor necrosis factor-alpha (TNF-α), and vascular endothelial growth factor A (VEGF-A). The choice of cytokines panel used in this study was based on the commercially available panel that contained the most known proinflammatory cytokines involved in canine atopic dermatitis [12, 15–18]. The kit was used according to the manufacturer’s instructions [25]. The MAGPIX platform (Luminex Corporation, TX, USA) was then used to read the plates, and the analyses were performed by the xPONENT v.4.2 software (Luminex Corporation, TX, USA) and Bio-Plex Manager v.6.1 software (BioRad Laboratories, Mississauga, ON, Canada). Evaluations of the quality control samples, standard curves, and bead counts were performed.

### Statistical analysis

Alpha diversity (number of genera and Chao, Simpson, and Shannon indices) was compared between the groups (healthy dogs versus untreated atopic dogs versus treated atopic dogs) with the analysis of variance (ANOVA) followed by the Tukey’s multiple comparisons test. A *P* value of < 0.05 was considered significant. Beta diversity (community membership and structure) was compared with the analysis of molecular variance (AMOVA) where a *P* value of < 0.05 was considered significant. Principal coordinate analyses were used to visualize the similarities between the different samples. Linear discriminant analysis effective size (LEfSe) was used to identify bacteria taxa significantly different between groups [26].

For cytokine concentrations, the non-parametric Kruskal-Wallis test was used to determine whether the distribution of the data varied among groups (healthy dogs versus untreated atopic dogs versus treated atopic dogs). When a significant difference was found, post hoc comparisons between pairs of groups adjusting the alpha level downward with the sequential Benjamini-Hochberg procedure were performed. A *P* value of < 0.05 was considered significant.

## Results

Bacterial microbiota analyses were performed on fifteen healthy dogs, fourteen untreated atopic dogs (AD), and six treated AD. The detailed results of the microbiota analysis of the anal sacs and rectum of the fifteen healthy dogs have been previously published [22]. Negative controls showed no presence of bacterial DNA. In the treated AD group, five and one dogs were treated with oclacitinib and desloratadine, respectively, and four dogs had been receiving ASIT concomitantly for at least nine months (Table 1).

### Relative abundances

**Fig 1:**
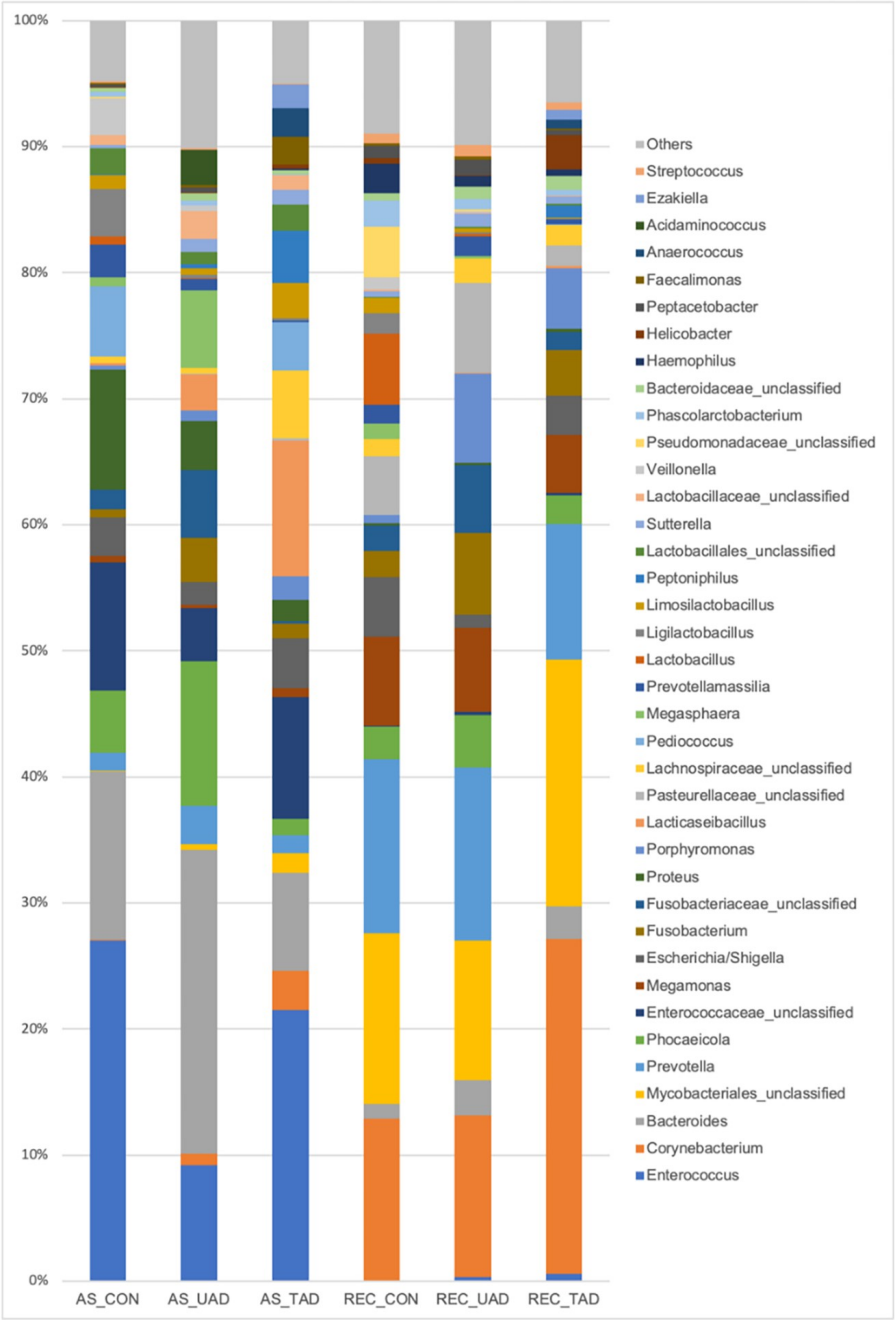
Mean relative abundance of the main bacterial genera (>1%) found in the anal sacs and rectum of healthy dogs and atopic dogs (untreated and treated). AS_CON, Anal sacs of healthy dogs; AS_UAD, Anal sacs of untreated atopic dogs; AS_TAD, Anal sacs of treated atopic dogs; REC_CON, Rectum of healthy dogs; REC_UAD, Rectum of untreated atopic dogs; REC_TAD, Rectum of treated atopic dogs.

Of the 28 phyla found (anal sacs and rectum combined), 694 different bacterial genera have been identified, but only 38 bacterial genera have a relative abundance of over 1% in the anal sacs and rectum of healthy dogs and untreated and treated AD (Fig 1). As shown in Fig 1, regardless of whether the dogs are atopic or not, the composition of the microbiota appears to differ between the anal sacs and rectum. The main bacterial genera found in the rectum of dogs in all groups were *Prevotella*, *Corynebacterium* and unclassified *Mycobacteriales*, while *Enterococcus* and *Bacteroides* were among the main genera found in the anal sacs of dogs.

**Fig 2:**
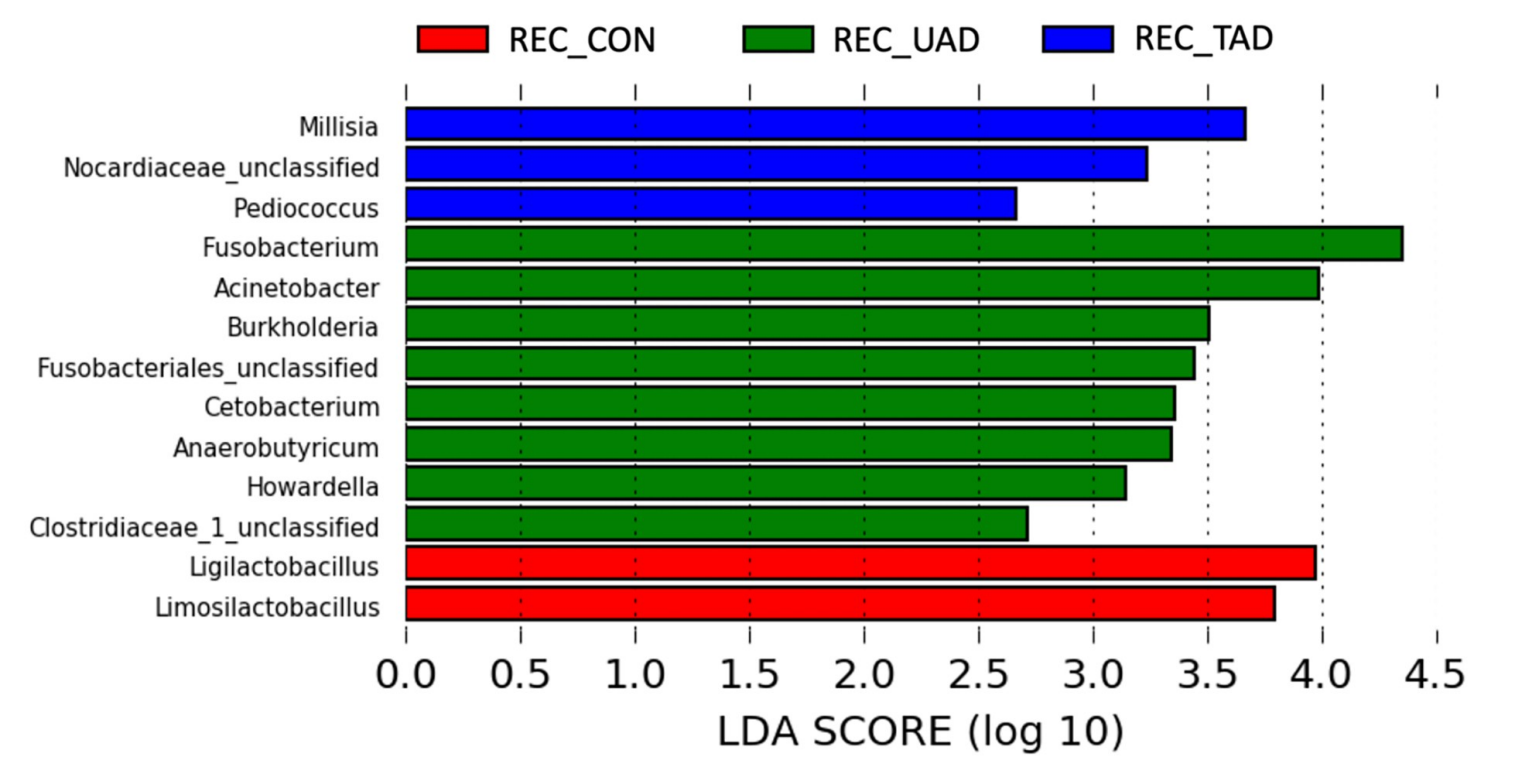
Linear discriminant analysis (LDA) effect size (LEfSe) indicating genera whose abundance is significantly different between the rectal microbiota in healthy dogs and atopic dogs (untreated and treated). REC_CON, Rectum of healthy dogs; REC_UAD, Rectum of untreated atopic dogs; REC_TAD, Rectum of treated atopic dogs.

Bacterial genera showing a significant difference in abundance between the rectum of healthy dogs, untreated AD, and treated AD assessed by the LEfSe analysis are presented in Fig 2. In addition, *Veillonella* spp. was overrepresented in the anal sacs of healthy dogs compared to the anal sacs of AD, while *Helicobacter* spp. and *Pasteurella* spp. were overrepresented in the anal sacs of treated AD (all *P* < 0.05).

### Alpha diversity

**Fig 3:**
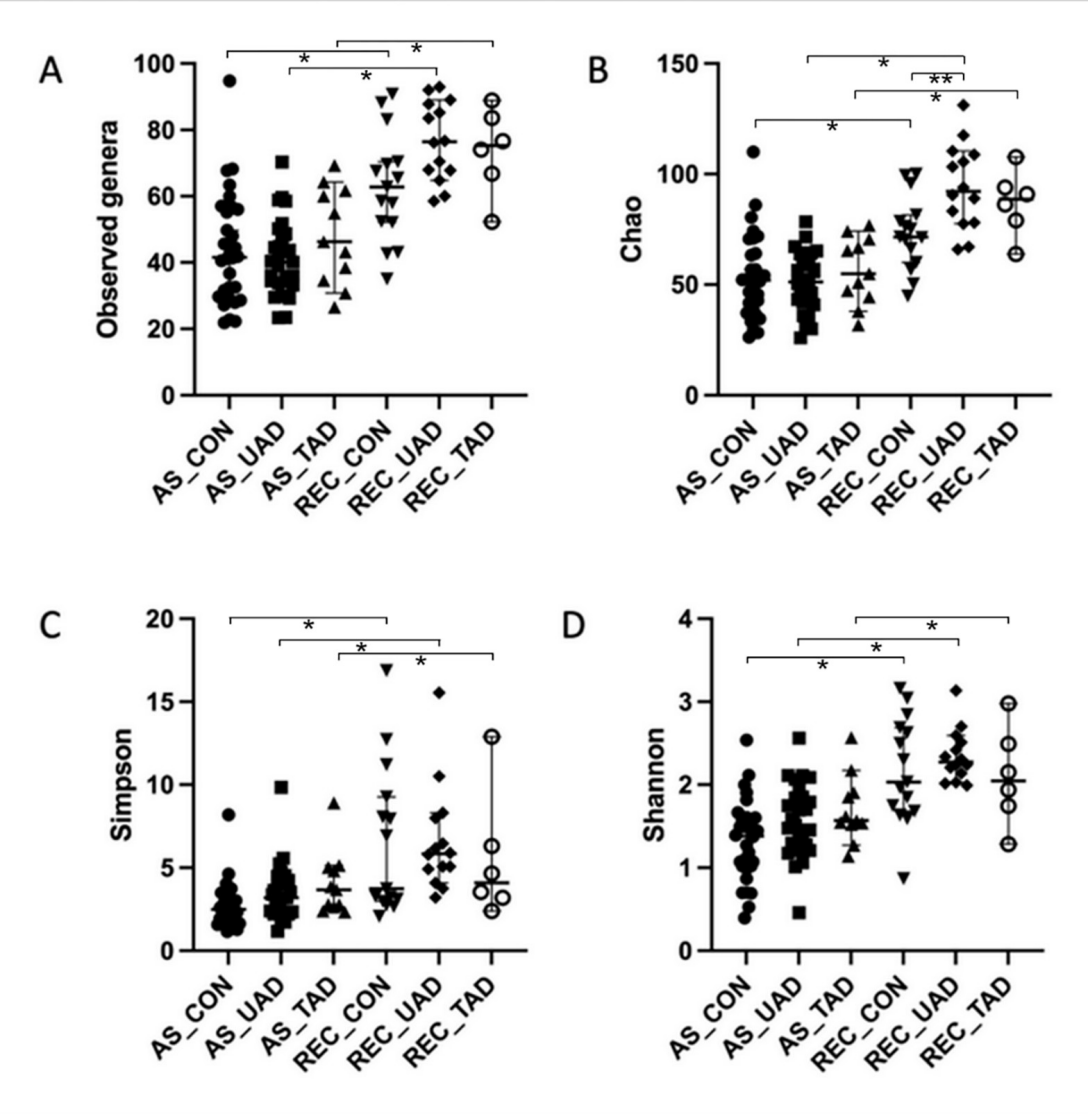
Alpha diversity measurements in different sites (anal sacs and rectum) in healthy dogs, untreated AD, and treated AD. A–number of genera observed. B–Chao index. C–Simpson index. D–Shannon index. * P < 0.001. ** *P* = 0.012. A *P* value lower than 0.05 was considered statistically significant. AS_CON, Anal sacs of healthy dogs, AS_UAD, Anal sacs of untreated atopic dogs; AS_TAD, Anal sacs of treated atopic dogs; REC_CON, Rectum of healthy dogs; REC_UAD, Rectum of untreated atopic dogs; REC_TAD, Rectum of treated atopic dogs.

For the alpha diversity analyses, one dog in the treated AD group was excluded because its left anal sac contents had a low sequence number. Overall, richness (number of bacterial genera found and Chao index) and diversity (Simpson and Shannon indices) were significantly higher in the rectum compared to anal sacs (*P* < 0.001 for all comparisons). The Chao index was significantly higher in the rectal samples from untreated AD compared to healthy dogs (*P* = 0.012), but no other significant differences were found in the comparisons between groups, including comparisons between anal sacs (Fig 3).

### Beta diversity

**Fig 4:**
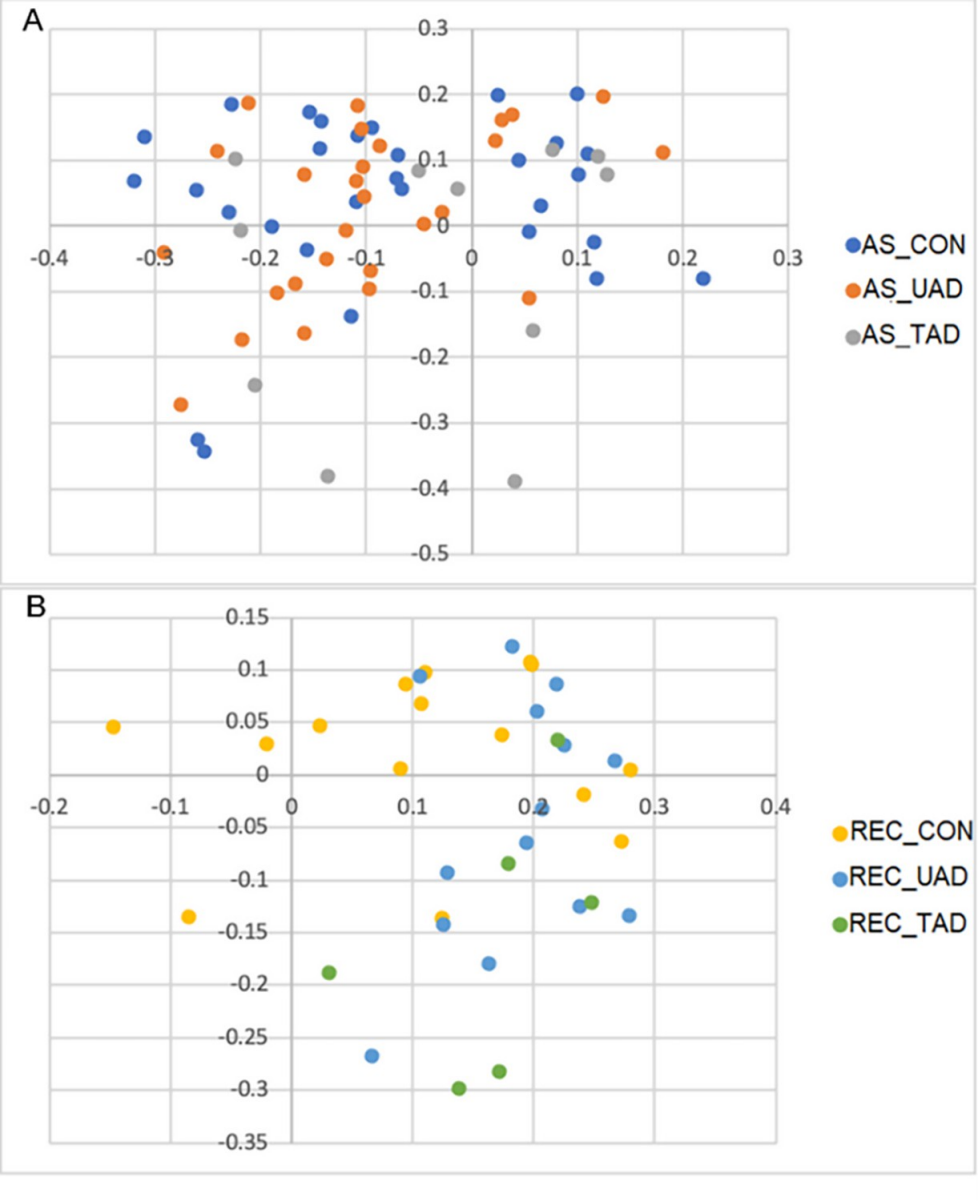
Principal Coordinate Analysis plots of the community membership comparing the anal sacs microbiota of healthy dogs, untreated AD, and treated AD (A) and the rectum microbiota of healthy dogs, untreated AD, and treated AD (B). AS_CON, Anal sacs of healthy dogs; AS_UAD, Anal sacs of untreated atopic dogs; AS_TAD, Anal sacs of treated atopic dogs; REC_CON, Rectum of healthy dogs; REC_UAD, Rectum of untreated atopic dogs; REC_TAD, Rectum of treated atopic dogs.

**Fig 5:**
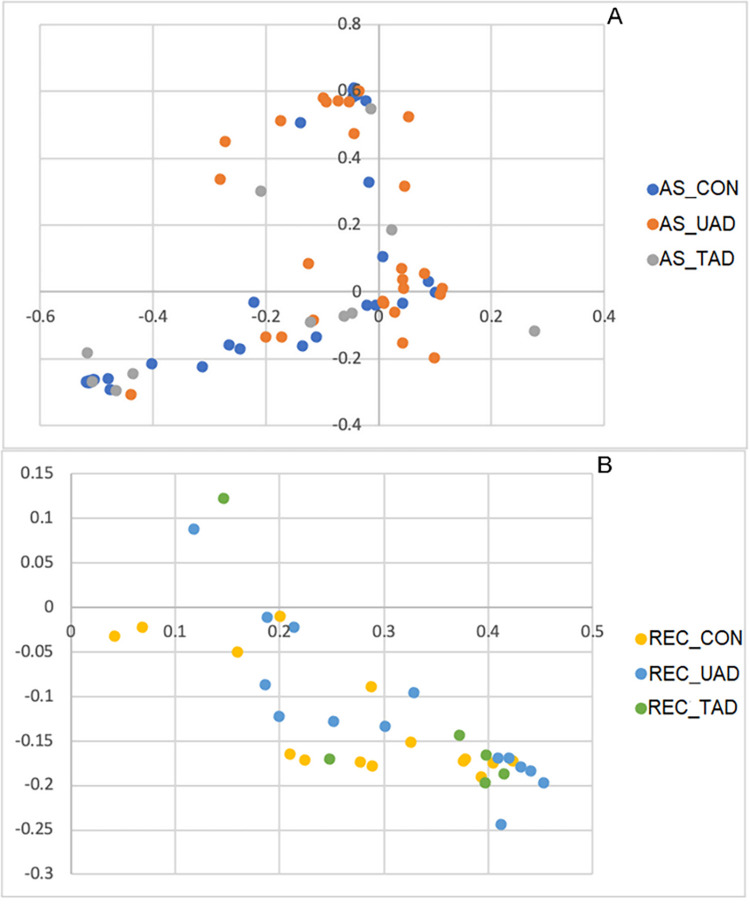
Principal Coordinate Analysis plots of the community structure comparing the anal sacs microbiota of healthy dogs with that of untreated and treated atopic dogs (A) and the rectum microbiota of healthy dogs with that of untreated and treated atopic dogs (B). AS_CON, Anal sacs of healthy dogs; AS_UAD, Anal sacs of untreated atopic dogs; AS_TAD, Anal sacs of treated atopic dogs; REC_CON, Rectum of healthy dogs; REC_UAD, Rectum of untreated atopic dogs; REC_TAD, Rectum of treated atopic dogs.

The community membership and community structure are represented by principal coordinate analyses (PCoA) in Figs 4 and 5, respectively, and *P*-values of statistical comparison of community membership and community structure between groups detailed in Table 2. The two-dimensional PCoA plots were able to explain 51% and 27% of data variation in membership and structure, respectively.

**Table 2:** P-values of statistical comparison of community membership and community structure between anal sacs and rectum of healthy dogs, and untreated and treated atopic dogs.

CM CS	Rectum of healthy dogs	Rectum of untreated AD	Rectum of treated AD	Anal sacs of healthy dogs	Anal sacs of untreated AD	Anal sacs of treated AD
Rectum of healthy dogs		**= 0.001**	**< 0.001**	**< 0.001**		
Rectum of untreated AD	= 0.460		**= 0.042**		**< 0.001**	
Rectum of treated AD	= 0.405	= 0.492				**< 0.001**
Anal sacs of healthy dogs	**< 0.001**				**= 0.002**	**= 0.013**
Anal sacs of untreated AD		**< 0.001**		**= 0.003**		**= 0.012**
Anal sacs of treated AD			**< 0.001**	= 0.332	**= 0.017**	

CM, Community membership; CS, Community structure; AD, Atopic dogs

*P* < 0.05 was considered significant and is indicated in bold.

The community membership differed between anal sacs and rectal microbiota within each group (*P* < 0.001). A difference in community membership was observed between the anal sacs of healthy dogs and untreated AD (*P* = 0.002), healthy dogs and treated AD (*P* = 0.013), and untreated AD and treated AD (*P* = 0.012). The community membership also differed between rectum of healthy dogs and untreated AD (*P* = 0.001), healthy dogs and treated AD (*P* < 0.001), and untreated AD and treated AD (*P* = 0.042).

The community structure differed between anal sacs and rectum of healthy dogs (*P* < 0.001), untreated AD (*P* < 0.001), and treated AD (*P* < 0.001). Dissimilarities were present in the community structure between the anal sac microbiota of healthy dogs and untreated AD (*P* = 0.003), as well as between untreated AD and treated AD (*P* = 0.017). However, the difference was not significant between the anal sac microbiota of healthy dogs and treated AD (*P* = 0.332), and between the rectal microbiota of healthy dogs and untreated AD (*P* = 0.460), healthy dogs and treated AD (*P* = 0.405), and untreated AD and treated AD (*P* = 0.492)

### Anal sac proinflammatory cytokine profile and concentrations

The proinflammatory cytokines were evaluated in fifteen healthy dogs, twelve untreated AD, and five treated AD. Two untreated AD and one treated AD were excluded from these evaluations because they did not have enough secretion in their anal sacs at the time of sampling. In addition, the contents of only one anal sac was collected and analyzed in four dogs (two healthy dogs and two treated AD), the contralateral anal sac containing not enough secretion when sampled.

The concentration of canine proinflammatory cytokines, including chemokines and growth factors, identified in the anal sacs of healthy and AD are summarized in Table 3. All proinflammatory cytokines tested (IFN-γ, IL-10, IL-8, IL-12/IL-23p40, IL-2, IL-6, TNF-α, MCP-1, NGF-β, SCF, and VEGF-A) were detectable in the anal sacs of healthy dogs and AD. There was no significant difference between healthy, untreated AD, and treated AD groups for all of them, except for IL-8 and TNF-α. The level of IL-8 was significantly higher in the anal sacs of untreated AD compared to treated AD (*P* = 0.02). The concentration of TNF-α was significantly lower in the anal sacs of healthy dogs compared to treated AD (*P* = 0.04).

**Table 3:** Cytokine concentrations (median and range) in pg/mL measured in anal sacs of healthy dogs, untreated atopic dogs, and treated atopic dogs.

Cytokines	Healthy dogs n = 15	Dogs with atopic dermatitis without treatment n = 12	Dogs with atopic dermatitis with treatment n = 5
IFN-g	4.25 (0.00–10.55)	6.97 (0.00–16.25)	8.32 (1.57–17.06)
IL-10	5.90 (0.00–21.57)	9.52 (1.12–18.53)	3.54 (1.44–18.53)
IL-12/IL-23p40	68.08 (20.73–458.89)	63.16 (35.03–165.70)	99.36 (23.09–381.51)
IL-2	12.32 (0.00–22.72)	11.34 (2.85–33.74)	10.49 (2.61–124.17)
IL-6	24.98 (10.71–44.43)	23.23 (11.05–43.05)	21.39 (14.25–41.23)
IL-8*	735.88 (84.77–30301.93)	**1991.24 (298.61–15278.21)**	**253.96 (0.00–859.05)**
MCP-1	164.30 (30.35–1629.70)	205.38 (61.97–2807.89)	348.83 (119.37–798.52)
NGF-β	2.55 (0.00–19.43)	9.74 (0.00–187.84)	10.37 (7.86–26.89)
SCF	22.87 (11.38–42.18)	27.89 (9.77–184.22)	19.55 (11.38–51.67)
TNF-α**	**6.85 (3.70–11.81)**	8.41 (2.59–110.28)	**9.44 (8.06–17.07)**
VEGF-A	7314.90 (2757.12–10989.15)	7210.52 (5015.85–8420.95)	7767.96 (6123.67–19339.63)

* Significant difference between untreated AD and treated AD (*P* = 0.02)

** Significant difference between healthy dogs and treated AD (*P* = 0.04)

## Discussion

This study used next-generation DNA sequencing (NGS) to show that the bacterial microbiota in the anal sacs of healthy dogs and atopic dogs is a rich and diverse environment. The main objective of the present study was to compare the anal sac microbiota between healthy dogs untreated AD, and treated AD. The significant difference observed in the composition of the bacterial microbiota of the anal sacs between healthy and untreated AD suggests a dysbiosis in the anal sacs of dogs with atopic dermatitis. This dysbiosis could favor the development of bacterial anal sacculitis in atopic dogs. Indeed, skin dysbiosis has been proposed as a major predisposing factor to bacterial skin infection in humans with atopic dermatitis [27, 28]. The fact that the community structure found in the anal sacs of untreated AD was different from that found in healthy dogs and treated AD, but that there was no difference between the community structure of the anal sacs of healthy dogs and treated AD, suggests that the treatments (oclacitinib, desloratadine and ASIT) administered to atopic dogs may have alleviated dysbiosis in the studied population.

The composition of the bacterial microbiota of the anal sacs and rectum was significantly different in all groups (healthy dogs, untreated AD, and treated AD). Considering that the anal sacs are a specific microenvironment, this difference could be explained by different biological or environmental factors, such as the type of glands, the presence of stool in the rectum, temperature, and humidity [1–3, 29]. In the present study, the composition of rectal microbiota also differed significantly between groups. This is consistent with the study by Thomsen et al. reporting a dysbiosis in the rectum of atopic dogs, and the study by Rostaher et al. in which the authors concluded that the faecal microbiota was different between healthy and atopic dogs [19, 30]. Although atopic dermatitis does not manifest with gastrointestinal signs, differences between the fecal microbiota in healthy humans and those with atopic dermatitis have also been reported, which may suggest that the intestinal microbiota has a role to play in the pathogenesis of atopic dermatitis in humans and dogs [31–35].

Healthy dogs had an overrepresentation of *Ligilactobacillus* spp. and *Limosilactobacillus* spp. in the rectum. Interestingly, in atopic humans the use of probiotics containing *Ligilactobacillus salivarius* (formerly *Lactobacillus salivarius*) has been reported to improve the clinical manifestation of the disease and the quality of life of people by modulating the gut microbiota and the immune system (stimulates and downregulates Th1 and Th2 response, respectively) [36–38]. Probiotics including *Limosilactobacillus reuteri* have been shown in turn to prevent the development of atopic dermatitis in the offspring, and to attenuate the clinical signs of atopic dermatitis in mice by modulating the immune response [39–41]. Dysbiosis affecting the abundance of *Ligilactobacillus* spp. and *Limosilactobacillus* spp. may therefore have a role to play in the pathogenesis of canine atopic dermatitis. It could be interesting to evaluate the effects of these probiotics on the development and clinical signs of atopic dogs as well as the impact on anal sacculitis.

Regarding the microbiota analyses, one of the limitations of this study was the use of a different DNA sequencing plate and lot of DNA extraction kits for healthy dogs and atopic dogs. It has been reported that these technical factors can impact the results of the composition of the bacterial microbiota [42–44], but we have observed consistent results from this sequencing center when using positive and negative controls to measure sequencing error.

The exact role played by the proinflammatory cytokines detected in the anal sacs of all groups in the development of anal sacculitis remains to be determined. It is noteworthy that to the best of the authors’ knowledge there are no studies published on cytokines released in the anal sacs of healthy or atopic dogs. In this study, it was not possible to determine which pro-inflammatory cytokines were likely involved in anal sacculitis in atopic dogs, as there was no significant difference in the concentration of most of the cytokines assessed between the three groups. We can hypothesize that these pro-inflammatory cytokines have no role to play in the development of anal sacculitis in the atopic dogs. It should be noted that several studies have reported divergent results for similar cytokines in the skin or peripheral blood of atopic dogs [12, 15, 16]. Further studies on anal sac contents or epithelium are therefore required to better understand the role of those proinflammatory cytokines in the anal sacs of atopic dogs.

A significant difference was detected between the two groups of atopic dogs with respect to IL-8, a pro-inflammatory chemokine mainly involved in neutrophil recruitment and activation in inflammatory sites [45]. Most treated AD were receiving oclacitinib (four of five dogs). Oclacitinib preferentially inhibits Janus kinase (JAK) 1, but also inhibits JAK2 and JAK3 to a lesser extent [46]. In humans, the JAK1/JAK2 inhibitor ruxolitinib has been shown to inhibit mast cell degranulation [47]. Upon degranulation, mast cells may release IL-8 [48]. In dogs, only one study evaluated the effect of oclacitinib on IL-8, but this study focused on the effect on mast cell tumor lines, which are not the ones involved in atopic dermatitis. The latter study showed that oclacitinib decreased the release of IL-8 from mast cell tumor lines *in vitro* [48]. Therefore, oclacitinib might affect IL-8 production and release in atopic dogs, reducing its expression in anal sacs of atopic dogs treated with this drug. The role of IL-8 in the anal sacs of atopic dogs is however unclear, since there was no significant difference between the anal sacs of healthy and untreated atopic dogs.

TNF-α, a predominantly pro-inflammatory cytokine [45], was elevated in treated AD when compared to healthy dogs in the present study. Some studies reported an increase of TNF-α mRNA expression in the skin or peripheral blood of atopic dogs compared to healthy individuals, while others reported no difference [12, 17, 49]. However, we would have expected either a higher concentration in the untreated AD compared to the healthy group or no difference between groups. This discrepancy could be explained by the low number of cases. Factors such as race, breed, and age, may also influence the composition and level of cytokines [50].

The small number of dogs enrolled in this study and the panel of cytokines tested are limitations that preclude a clear relationship between cytokines, atopic dermatitis, and a predisposition to bacterial anal sacculitis. Another limitation of this study was the use of the ProcartaPlex panel with the Luminex xMAP technology, which is designed to assess the canine cytokines in serum, plasma, or cell culture supernatants [25]. The use of anal sac contents may therefore have affected the sensitivity and specificity of the test. With the plate used, only eleven proinflammatory cytokines were evaluated. The use of a more specific with higher throughput methodology, such as the OLINK high-throughput proteomic platform used in human medicine to assess thousands of proteins simultaneously, would have been helpful but much more expensive [51]. Another limitation of the present study was the assessment of cytokines from anal sac secretions. It is possible that cytokines of importance in atopic dermatitis were not detected if, for example, they were not released from the epithelium in the secretions of anal sacs. Biopsies of the anal sacs and measurement of cytokines within the epithelium would have been interesting, but more invasive with higher risks of complications.

## Conclusion

There is a dysbiosis in the anal sacs of atopic dogs. It is unclear whether dysbiosis is secondary to the inflammation driven by atopic dermatitis, or inherent to atopy itself. The treatments (oclacitinib, desloratadine, or ASIT) shifted the anal sac microbiota toward those of healthy dogs. The anal sacs of healthy and atopic dogs contain several proinflammatory cytokines. Amongst them, IL-8 might play a role in the development of disease and deserves further evaluation. The results of this study suggest that a dysbiosis may contribute to the development of bacterial anal sacculitis in dogs suffering from atopic dermatitis. Further studies on a larger number of dogs, evaluating the effect of a wider range of antipruritic drugs (e.g. glucocorticoids, cyclosporine, lokivetmab) and proinflammatory cytokines would be required for a better understanding of the pathogenesis of anal sacculitis in atopic dogs, which could eventually lead to the development of new therapeutic approaches or preventive measures to anal sacculitis.
